# High prevalence of *KRAS* and *GNAS* mutations in pseudomyxoma peritonei underscores opportunities for targeted therapeutic strategies

**DOI:** 10.1515/pp-2025-0034

**Published:** 2025-12-15

**Authors:** Annette Torgunrud, Christin Lund-Andersen, Ben Davidson, Ina Katrine Nitschke Marcussen, Stein G. Larsen, Vegar Dagenborg, Kjersti Flatmark

**Affiliations:** Department of Tumor Biology, Norwegian Radium Hospital, Oslo University Hospital, Oslo, Norway; Department of Pathology, Oslo University Hospital, Oslo, Norway; Medical Faculty, Institute of Clinical Medicine, University of Oslo, Oslo, Norway; Department of Surgical Oncology, Norwegian Radium Hospital, Oslo University Hospital, Oslo, Norway

**Keywords:** pseudomyxoma peritonei, *KRAS *mutation, *GNAS *mutation, oncogenes, targeted therapy

## Abstract

**Objectives:**

Pseudomyxoma peritonei (PMP) is a rare, slow-growing cancer with few efficacious treatment options in unresectable cases. Mutations in the *KRAS* and *GNAS* oncogenes are common, but the reported frequencies vary greatly, most likely because of low tumor cellularity in peritoneal tumor samples. With treatments targeting these mutations becoming increasingly available, reliable detection of mutations is essential.

**Methods:**

The frequency of *KRAS* and *GNAS* mutations was analyzed in tumor samples from 167 patients with verified PMP using targeted DNA sequencing and/or droplet digital polymerase chain reaction. When analysis of fresh-frozen peritoneal tumor samples did not reveal mutations, macrodissected formalin-fixed samples were analyzed.

**Results:**

Mutations in cancer-related genes were detected in 98 % of the analyzed samples, with *KRAS* and *GNAS* mutated in 148 (89 %) and 139 (83 %) cases, respectively. In 48 % of the analyzed cases, the mutational diagnosis was based on primary tumor samples.

**Conclusions:**

High frequencies of *KRAS* and *GNAS* mutations support the proposed role as driver mutations and as potential therapy targets. The primary tumor may serve as an alternative source of tumor material, increasing the likelihood of detecting targetable mutations. Combined with highly sensitive analytical methods, this approach facilitates selection of patients for novel targeted therapeutic strategies.

## Introduction

Pseudomyxoma peritonei (PMP) is a rare, progressive, but generally slow-growing abdominal cancer with an incidence rate of 3.2 people per million per year [1]. It is commonly caused by ruptured appendiceal mucinous neoplasms seeding tumor cells and mucin into the peritoneal cavity. Standard-of-care treatment involves cytoreductive surgery (CRS) to remove all visible tumor tissue, followed by hyperthermic intraperitoneal chemotherapy (HIPEC) to eliminate microscopic residual disease [2], [3], [4]. This treatment is curative in approximately 50 % of the patients, but for patients who cannot be cured by CRS-HIPEC, few efficacious treatment options exist. In unresectable and recurrent PMP, mucinous tumor tissue will gradually accumulate in the abdomen, representing a significant burden on patients and a huge therapeutic challenge for health care personnel responsible for their care. Developing new therapies for PMP is, therefore, an important research priority, and improved knowledge concerning the molecular features of the disease is essential to identify potential actionable therapeutic targets.

Mutations in the *KRAS* and *GNAS* oncogenes have been assumed to be key molecular drivers of PMP, but the reported frequencies of these mutations are highly variable [5], 6]. The lowest detection frequencies of *KRAS* and *GNAS* mutations were 38 % and 17 %, respectively [7], 8], with other studies reporting mutations in up to 100 % of cases [9]. An important reason for the variable results is that tumor samples from the peritoneal disease in many cases have a very low tumor cell to mucin ratio, making identification of mutations challenging. With therapies directly or indirectly targeting these aberrations becoming increasingly available to cancer patients, it is becoming important to know how frequently these mutations occur in PMP, and to have reliable analytical strategies to identify individual cases that might benefit from such treatment [10], [11], [12].

In this work, we have analyzed the frequency of *KRAS* and *GNAS* mutations in peritoneal metastases and primary tumors from a large cohort of PMP cases using highly sensitive molecular methods, aiming to establish the frequency of these mutations and analyze their associations with clinicopathological parameters.

## Materials and methods

### Patients

Patients undergoing surgery for suspected PMP at the Norwegian Radium Hospital, Oslo University Hospital as part of the national multimodal treatment program for peritoneal surface malignancies were eligible for inclusion. The study was approved by the Regional Ethics Committee of South-Eastern Norway (Project ID# s-07160b), and written informed consent was required for participation. The study was performed in accordance with the Declaration of Helsinki. Race and ethnicity data were not collected or recorded for this cohort and could, therefore, not be reported in the participant demographics. Of 296 patients undergoing surgery between September 2002 and June 2022, 196 were included in the study with the intention to collect tumor tissue, the majority after 2013, when routine biobanking was initiated (Supplementary Figure S1). Of the 196 included patients, 29 were excluded from analysis, leaving a study cohort of 167 patients: 11 patients had appendiceal tumors but no PMP; in 17 patients, tumor was not collected at the time of surgery, and no suitable formalin fixed paraffin embedded (FFPE) sections were available; for one patient, insufficient DNA was extracted from the sample. Clinical data were retrieved from the institutional peritoneal surface malignancy database. Peritoneal tumor distribution was classified using the peritoneal cancer index (PCI), giving a score between 0 and 39 [13], and residual tumor after CRS was classified using the completeness of cytoreduction (CC) score (CC-0, no residual tumor; CC-1, residual tumor <0.25 cm; CC-2, tumor between 0.25 and 2.5 cm; CC-3, tumor >2.5 cm) [13]. Mitomycin C (MMC)-based HIPEC (35 mg/m^2^ over 90 min, was administered with 50 %/25 %/25 % every 30 min) when complete cytoreduction was defined as CC-0 and CC-1. Tumor marker analysis of carcinoembryonic antigen (CEA), cancer antigen 19-9 (CA19-9), and cancer antigen 125 (Ca-125) at the time of surgery was available for 132, 128, and 166 patients, respectively.

### Tumor sample processing

Tumor samples collected at the time of surgery were immediately snap frozen in liquid nitrogen and stored at −80 °C until further processing. Between one and four tissue aliquots from each patient were sectioned and stained to determine tumor content in hematoxylin and eosin-stained frozen sections. The proportion of tumor cells in the samples was semi-quantitatively assessed (no tumor, <1 %, 1 %–10 %, 10 %–50 %, and >50 %; Supplementary file, Table S1). The sample with the highest tumor content was chosen for further processing when possible; from assumed “acellular” samples, one random tissue aliquot was analyzed. Samples were homogenized and disrupted using TissueLyzer LT (Qiagen, Hilden, Germany), and DNA was extracted using the AllPrep DNA/RNA/miRNA Universal Kit, automated with the use of QIAcube (Qiagen). If fresh frozen tissue was not available or if no mutations were detected in DNA extracted from fresh frozen tissue samples, FFPE routine pathology samples from the peritoneal or primary tumor were obtained, examined for the presence of tumor tissue, and macrodissected to obtain representative samples. DNA was extracted using the AllPrep DNA/RNA FFPE Kit (Qiagen). For all samples, DNA purity was measured using NanoDrop (Thermo Fisher Scientific, Waltham, MA, USA), and DNA concentrations were determined with the Qubit fluorometer (Thermo Fischer).

### Targeted DNA sequencing and ddPCR

Targeted next-generation sequencing (NGS) was performed with the PGM/Ion GeneStudio S5 system and the Ion AmpliSeq™Cancer Hotspot Panel v2 covering 50 unique genes. The median variant allele sequencing depth was 3,765, enabling detection down to 1 % of sequenced reads matching a specific DNA variant. Variants were called, annotated, and filtered with Ion Reporter Software 5.10 (Thermo Fisher). The following filtering criteria were set to minimize inclusion of false positive and germline variants; synonymous, UCSC common SNPs, MAF > 0.002, likely benign/benign in the ClinVar database, phred score <20 and p-value >0.05. All reported variants were manually reassessed using Integrative Genomics Viewer. In parallel, 28 samples were analyzed using the Oncomine Comprehensive Assay v3 (Thermo Fischer), covering single-nucleotide variants (SNVs) and indels from 161 unique genes with the filtering criteria; UCSC common SNPs, MAF > 0.002, likely benign/benign in the ClinVar database, phred score <20 and p-value >0.05.

In samples where *GNAS* and *KRAS* mutations were suspected based on NGS, but below the threshold value of 1 % mutated allele frequency or read counts <20 for the mutant variant, validation was performed using the ddPCR system from BioRad (BioRad^®^, Hercules, CA, USA). The following assays were used: ddPCR™ Mutation Assay (*GNAS* p.R201H (assayID: dHsaMDV2516796), *GNAS* p.R201C (assayID: dHsaMDV2510562), *KRAS* p.G12D (assayID: dHsaMDV2510596), *KRAS* p.G12V (assayID: dHsaMDV2510592), *KRAS* p.G13D (assayID: dHsaMDV2510598), and *KRAS* G12/G13 (Scr kit catalog #1863506). Premix preparation, droplet generation, and thermal cycling were performed according to the manufacturer’s instructions. The fluorescence intensity in droplets was detected by a QX200 Droplet Reader (BioRad). For all assays, a “no template control” and a positive control were included for quality control. QuantaSoft version 1.7.4 analysis software and QX Manager Software (BioRad) was used for data acquisition and analysis. Only tests providing >13.000 droplets were considered valid, and for the mutation to be verified as present, a minimum of three positive droplets with mutated DNA fragment was needed.

### Histopathological classification

Routine histological sections were evaluated by the study pathologist (BD), and the primary appendiceal tumor and peritoneal disease were classified according to the Peritoneal Surface Oncology Group International (PSOGI) classification [14]. For subsequent statistical analyses, the primary appendiceal tumors were classified into two groups: the low-grade appendiceal mucinous neoplasm group (LAMN group) and the non-LAMN group, comprising high-grade appendiceal mucinous neoplasms (HAMN), mucinous adenocarcinomas, and goblet cell carcinoids.

### Statistical analysis

Variables are described with percentages or medians (min-max). CC-score was used to classify patients into two groups: CC-0 and CC-1 (CC ≤ 1 group), which was defined as complete cytoreduction, and CC > 1 group, which included CC-2 and CC-3. Variables in these subgroups were compared using Chi-square test for frequencies and Mann–Whitney U test to compare medians. Time was described in months. Overall survival (OS) was defined as the time from the first procedure with the intention to perform CRS (index operation) to the date of death (from the Norwegian National Population Registry) or the censor date (January, 31st, 2024). Disease-free survival (DFS) was defined as the time from the index operation to the first recurrence, the last date of radiological imaging or death. Univariable analyses were performed by the Kaplan–Meier method to estimate OS and DFS and compared by log-rank. The reverse Kaplan–Meier method was used to estimate median follow-up time and median time to event. Hazard ratios (HR) were calculated using Cox proportional hazard analysis and were reported with 95 % confidence interval (CI). p-values <0.05 were considered statistically significant. Multivariable analyses were performed by Cox proportional hazard analysis for age and gender in addition to variables with a p-value <0.1 from univariable analyses. The clinical data were analyzed with SPSS statistics (version 29.0.0.0 (241), IBM Corp, Armonk, NY).

## Results

### Patients, procedures, and histopathological classification

Of the 167 study patients, 105 were female (63 %), and the median age was 56 years (22–81 years). The median PCI score for the total cohort was 25, and the majority of the patients had complete cytoreduction, CC ≤ 1 (n = 135; 81 %). The patients in the CC ≤ 1 group had less extensive peritoneal disease than the CC > 1, with median PCI scores of 22 and 35, respectively (p = 0.007) (Table 1). All except two patients in the CC ≤ 1 group received HIPEC (one patient died perioperatively of complications; one patient received early postoperative intraperitoneal chemotherapy (EPIC)).

**Table 1:** Clinical and pathological parameters of the study cohort.

	All patients n = 167	CC ≤ 1 n = 135	CC > 1 n = 32
Median age (min–max)	56 (22–81)	55 (22–78)	63 (35–81)
Sex (%)			
Female	105(63)	86(54)	19(59)
Male	62(37)	49 (36)	13(41)
Peritoneal tumor distribution			
Median PCI (min–max)	25 (0–39)	22 (0–39)	35 (14–39)
PCI 0–10 (%)	26 (16)	26 (19)	0
PCI 11-20	34 (20)	32 (24)	2 (6)
PCI 21-30	50 (30)	45 (33)	5 (16)
PCI >30	57 (34)	32 (24)	25 (78)
Median tumor markers (min-max)			
CEA	6 (0–671)	5 (0–671)	38 (1–55)
CA19-9	18 (0–34,223)	11 (0–853)	152 (4–34,223)
CA-125	32 (4–698)	23 (4–395)	80 (13–698)
Primary tumor location (%)			
Appendix	145 (87)	122 (90)	23 (72)
Ovary	4 (2)	4 (3)	0
Unknown	18 (11)	9 (7)	9 (28)
Histological classification appendix tumor (%)			
LAMN	121 (73)	106 (79)	15 (47)
Non-LAMN group			
HAMN	13 (8)	9 (7)	4 (13)
Mucinous adenocarcinoma	9 (5)	6 (4)	3 (9)
Goblet cell carcinoid	1 (0.6)	0	1 (3)
NA	1 (0.6)	1 (0.7)	0
Histological classification peritoneal disease (%)			
LG	117 (70)	96 (71)	21 (66)
Acellular mucin	28 (17)	28 (21)	0
HG	19 (11)	11 (8)	8 (25)
HG with signet ring cells	3 (2)	0	3 (9)

CC, completeness of cytoreductive surgery score; PCI, peritoneal cancer index; LAMN, low-grade appendiceal mucinous neoplasm; HAMN, high-grade appendiceal mucinous neoplasms; NA, not available; LG, low-grade; HG, high-grade.

The location of primary tumor was in the appendix in 145 (87 %) of the study patients; four patients (2 %) had primary ovarian tumors, and in 18 patients (11 %), the primary tumor location was unknown. The appendiceal tumors were classified as LAMN in 121 cases (73 %), HAMN in 13 (8 %), mucinous adenocarcinomas in 9 (5 %), one patient had a goblet cell carcinoid, and in 1 case, the appendix tumor was not available for classification. The peritoneal disease was classified as low-grade in 117 cases (LG; 70 %), acellular mucin without identifiable neoplastic epithelial cells in 28 (17 %), high-grade in 19 (HG; 11 %), and HG with signet ring cells in 3 (2 %) (Table 1). In three of the LAMN cases, HG peritoneal disease was diagnosed, while conversely, nine cases with non-LAMN primary disease presented with LG peritoneal disease. Most of the patients with LG/acellular mucin peritoneal disease (100/121, 83 %) had complete cytoreduction, compared to only half of the patients with HG/HG with signet ring cells peritoneal disease (11/22, 50 %) (p < 0.001).

### DNA mutation analysis

Mutations in cancer-related genes were found in samples from 164 of the 167 study patients (98 %). The most frequent mutations were detected in *KRAS*; in 148 cases (89 %) followed by *GNAS* in 139 cases (83 %). The most frequent *KRAS* aberrations were missense mutations located at the two major hot spots in codon 12 (p.G12D (n = 67; 40 %), p.G12V (n = 43; 26 %), p.G12C (n = 9; 5 %)) and codon 13 (p.G13D (n = 22; 13 %)), and six patients (4 %) had other *KRAS* mutations. The most frequent *GNAS* mutations were located at codon 201, R201H (n = 95; 68 %) and R201C (n = 39; 28 %), while other *GNAS* mutations were found in five patients (4 %). *KRAS* and *GNAS* mutations co-occurred in 130 cases (78 %). Of the three patients where no mutations were detected, two had LAMN primary histology, one had a mucinous appendix adenocarcinoma, and all had LG peritoneal disease.

**Figure 1:**
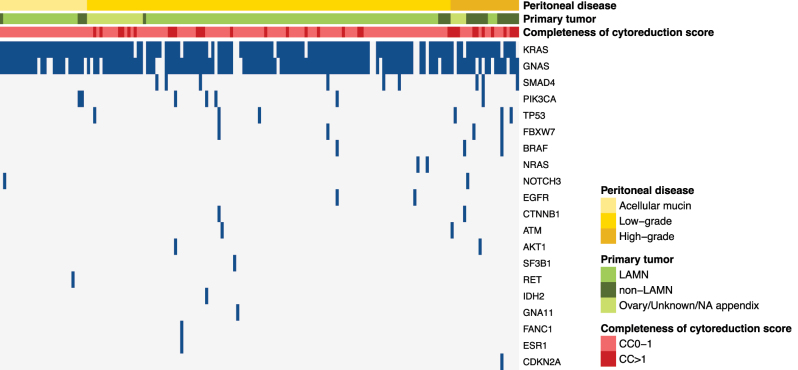
Oncoplot showing mutations grouped according to histological classification of the peritoneal disease, also including information on primary tumor, and the completeness of cytoreduction score. LAMN low-grade appendiceal mucinous neoplasm, HAMN high-grade appendiceal mucinous neoplasm, NA not available.

Mutations in genes other than *KRAS* and *GNAS* were detected in 35 (21 %) of the patients, of which 28 also had mutations in *KRAS* and/or *GNAS* (Table S1). In cases analyzed with a broader 161 gene panel (Oncomine v3), only four additional mutations were detected (not covered by the 50 gene panel) in three cases (Table S2). The most commonly mutated genes other than *KRAS/GNAS* were *SMAD4* (9/35), *PIC3CA* (7/35), *TP53* (6/35), *FBXW7* (4/35), and *BRAF* (3/35) (Figure 1 and Table S2). Mutations in genes other than *KRAS* and *GNAS* were more frequently detected in patients with non-LAMN primary appendix tumors (10/23 cases; 44 %) and HG/HG with signet ring cell peritoneal disease (11/22 cases; 50 %) compared to patients with LAMN primaries (21/121; 17 %) and LG/acellular mucin peritoneal disease (24/145 cases; 17 %); p < 0.005 and p < 0.001 for comparisons, respectively.

### Tissue basis for mutational diagnoses

Of the 164 cases with detected mutations, 86 conclusions were based on results obtained from fresh-frozen peritoneal tumor samples. Interestingly, in 21 of these cases with detected mutations, no tumor cells had been identified on microscopy of the frozen sections. Of the cases with no visible tumor upon microscopy of frozen sections, 21 cases were classified as acellular mucin by routine pathology, while 49 were low-grade and one was high-grade. Mutations were detected by analysis of these fresh-frozen peritoneal samples in 21 of these 71 cases; of these, three were classified as acellular mucin. For the remaining 78 cases, microdissection of FFPE sections was necessary to obtain tumor DNA. Sections were obtained from the primary appendix tumor or the peritoneal disease in 53 and 25 cases, respectively. In six cases, where matched, verified tumor tissue was available from both the primary and peritoneal tumor, identical mutations were detected.

### Long-term outcome and associations with clinical and mutational data

**Figure 2:**
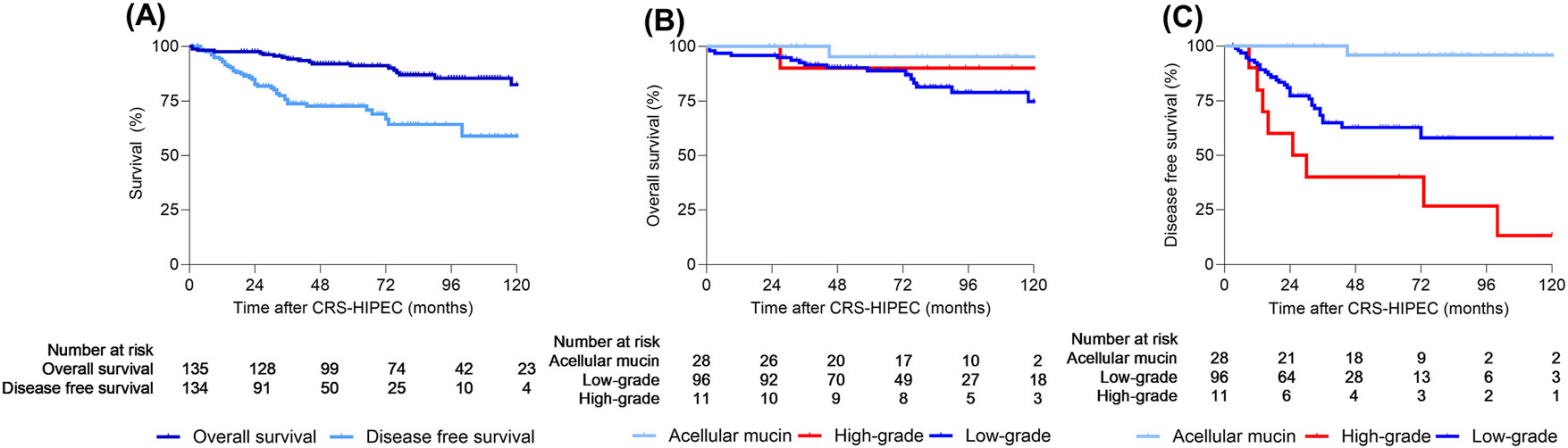
Kaplan–Meier curves showing overall and disease-free survival in patients undergoing complete cytoreductive surgery and hyperthermic intraperitoneal chemotherapy (CRS-HIPEC). (A) Overall and disease-free survival. (B) Overall survival according to histopathological classification of the peritoneal disease component. (C) Disease-free survival according to histopathological classification of the peritoneal disease component.

Forty-three patients died during follow-up (22 of 135 in the CC ≤ 1 group and 21 of 32 in the CC > 1 group). The median follow-up time after surgery for the CC ≤ 1 group was 83 months (95 % CI, 77–89 months). The median OS was not reached, and the estimated 5- and 10-year OS were 92 % and 80 %, respectively. For the CC > 1 group the estimated median 5- and 10-year OS were 37 % and 18 %, respectively (Figure 2a). In the CC ≤ 1 group, one patient died perioperatively, leaving 134 patients for assessment of DFS. Peritoneal recurrences were detected in 38 of the 134 patients (28 %), with a median DFS follow-up time of 49 months (95 % CI, 35–63 months) and 5- and 10-year DFS of 68 % and 55 %, respectively (Figure 2a). Three patients were diagnosed with additional cancer (melanoma, sarcoma, and primary lung cancer).

Patients diagnosed with acellular mucin had 95 % 10-year OS, and none of the 28 patients had a recurrence during 10 years of follow-up. Patients with LG disease had 5- and 10-year OS of 89 % and 75 %, respectively, while 5- and 10-year DFS were 62 % and 56 %, respectively (Figure 2b and c). Interestingly, the 11 patients with HG peritoneal disease that obtained complete cytoreduction also had excellent 10-year OS (90 %), and only one of these patients died during follow-up (Figure 2b), while the 5- and 10-year DFS were 40 % and 13 %, respectively (Figure 2c). There were no associations between the presence of *KRAS* and *GNAS* mutations and long-term outcome. Increasing PCI, presence of *SMAD4* mutation, and elevated CA19-9 and CA-125 were associated with inferior OS (Table 2), while CA19-9 was the only significant parameter remaining associated with worse OS in multivariable analysis with HR of 1.0 (95 % CI:1.00–1.01), p=0.013 (Table S3).

**Table 2:** Univariable analysis of parameters associated with overall survival.

	HR (95%CI)	p-Value
Sex		
Male	Reference	
Female	1.99 (0.85–4.69)	0.12
Age		
Increasing	1.01 (0.97–1.04)	0.63
Peritoneal cancer index		
Increasing	1.03 (1.03–1.17)	0.01
Eastern cooperative oncology group		
ECOG 0	Reference	
ECOG 1	3.35 (0.85–13.20)	0.08
ECOG 2	4.92 (0.60–40.26)	0.14
Histological classification appendix tumor		
LAMN group	Reference	
Non-LAMN group	0.04 (0.0–19.98)	0.31
Histological classification peritoneal disease		
Acellular mucin	Reference	
Low-grade	5.03 (0.67–37.81)	0.12
High-grade	1.60 (0.97–26.22)	0.74
Mutations		
*KRAS*	0.84 (0.25–2.86)	0.78
*GNAS*	0.82 (0.28–2.44)	0.71
*SMAD4*	4.21 (1.21–14.65)	0.02
*PIC3CA*	0.05 (0.00–2,335.89)	0.58
Tumor markers		
CEA	1.00 (0.10–1.01)	0.31
CA19-9	1.00 (1.00–1.01)	0.01
CA-125	1.01 (1.01–1.02)	<0.001

CEA, carcinoembryonic antigen; CA19-9, cancer antigen 19-9; Ca-125, cancer antigen 125.

## Discussion

The main finding after mutational analysis of tumors from this large cohort of PMP patients was a high frequency of mutations in the *KRAS* and *GNAS* oncogenes. The frequencies of 89 % and 83 %, respectively, were in the high range of what has been reported previously [6]. The reported variability from previous studies could be explained by random effects in small patient cohorts, heterogeneity in patient selection and tissue sampling, and varying sensitivity of the applied technologies. A recent report from a large UK cohort [15] employing NGS analysis of fresh-frozen peritoneal samples from 223 PMP patients reported mutations in 56.5 % of the analyzed samples. This is almost identical to our findings of 58 % mutated samples if we only consider results from the fresh-frozen peritoneal tumor samples. The frequency of *KRAS* and *GNAS* mutations in the UK cohort was 42 % for both genes, while we found 51% and 48 %, respectively, in the fresh-frozen peritoneal samples. However, upon interrogating DNA isolated by microdissection of formalin fixed tissues from peritoneal tumors or the corresponding primary, mutations were detected in almost all cases in our cohort. This suggests that the primary tumor could be an alternative source for determining the mutational status in PMP cases with low tumor cell content in the peritoneal disease component. In six patients, available parallel samples from the primary appendix tumor and peritoneal disease showed identical mutations in *KRAS* and *GNAS*, which is consistent with previous reports [5], 16]. The high frequency of these mutations suggests that both *KRAS* and *GNAS* mutations are essential for PMP development and progression, most likely representing driver mutations. Furthermore, our preliminary findings of identical mutations in the primary tumor and peritoneal disease in six of the analyzed cases are in line with previous reports and indicate that either the primary tumor or the peritoneal disease component could be analyzed to identify mutations.

Mutations other than *KRAS* and *GNAS* were detected in 21 % of the samples. Of these, the most interesting findings were mutations in *SMAD4*, which were associated with inferior outcome, and *BRAF* mutations, which could be targeted by BRAF inhibitors. A higher frequency of mutations other than *KRAS* and *GNAS* was detected in HG cases (HG or HG with signet ring cells) compared to LG cases (LG or acellular mucin). The low detection rate of other mutations could in part be attributed to the relatively limited coverage of the gene panels used. Studies where broader panels or exome sequencing were applied have reported a plethora of mutational findings, while the mutational burden has been reported to be very low [9], 17]. On the other hand, the panels used in this study were designed to cover the most commonly mutated cancer genes and provide decision support for the most common targeted treatments. One might, therefore, question the need for extensive sequencing coverage, except for exploratory purposes.

In this study, the presence of *KRAS* and *GNAS* mutations was not associated with long-term outcomes. Previous research have reported inconsistent results, one in agreement with our findings [18], while others have found that *KRAS* and/or *GNAS* mutations in PMP were associated with inferior oncological outcomes [15], [17], [18], [19], [20], [21]. These studies had higher proportions of HG tumors (up to 63 %), compared to our cohort where only 13 % of cases were HG. Microscopically, the peritoneal tumor component of HG tumors tends to have a higher tumor to mucin ratio than LG tumors, which could lead to high detection frequency of mutations in peritoneal tumors. Since HG histology is also associated with inferior prognosis, the proportion of HG cases when only peritoneal tumors are analyzed could confound the interpretation of associations with prognostic variables.

The high prevalence of *KRAS* and *GNAS* mutations in PMP underscores their relevance as targets for molecular therapy. The search for clinically relevant small molecule inhibitors of mutated *KRAS* has so far resulted in approval of selective KRAS^G12C^ inhibitors for clinical use [11], 12], 22]. Based on our results, a small proportion of PMP patients (5 %) could expect to benefit from these inhibitors. With the success of the KRAS^G12C^ inhibitors, extensive efforts to develop other mutant-selective inhibitors as well as pan-RAS inhibitors are ongoing [23], [24], [25], [26]. Of particular interest, the *KRAS* G12D inhibitor MRTX1133 has shown promising results in a high-grade PMP xenograft mouse model and could be clinically interesting with G12D being the most common mutation, identified in 40 % of our cases [27]. Similarly, the EFTX-G12V EGFR-targeted RNAi strategy is shown to selectively silence *KRAS* G12V, which was detected in 26 % of our patients [28]. Targeting mutated *GNAS* has so far not been successful, but the high frequency of mutations in codon 201 in PMP makes this gene an interesting target. The mutation causes constitutive activation of downstream signaling through the protein kinase A pathway, which may explain the massive production of mucin and suggests that mutated *GNAS* is a major oncogenic driver in PMP [7]. An interesting approach could be to target the *GNAS* mutation indirectly using the cyclin-dependent kinase 4/6 inhibitor palbociclib, which was shown to exhibit antiproliferative effects and clinical efficacy in *GNAS*-mutated appendiceal cancer [29]. Furthermore, we previously demonstrated that patients with *GNAS*-mutated PMP have a preexisting, attenuated immune response against mutated *GNAS* [30]. Based on this finding, we are initiating the Pseudovax trial, aiming to explore treatment with mutated peptide combined with immune checkpoint inhibition (ICI), hoping to reactivate the immune response and restore measurable anticancer T-cell immunity. Taken together, novel therapies targeting *KRAS* and *GNAS* mutations directly or indirectly can be expected to become available in the near future, and it is, therefore, important to be able to identify individual cases that might benefit from such targeted treatment.

## Conclusions

High frequencies of mutations in the *KRAS* and *GNAS* oncogenes were demonstrated in this large PMP cohort. The primary tumor may serve as an alternative source of tumor tissue if mutations are not detected in the peritoneal disease component. With the increasing availability of treatments targeting *KRAS* and *GNAS* mutations, the results further underline the importance of accurate identification of mutations to identify treatment opportunities for PMP patients.

## Supplementary Material

Supplementary Material
