# Supplementary material for: High prevalence of KRAS and GNAS mutations in pseudomyxoma peritonei underscores opportunities for targeted therapeutic strategies
Source: Pleura Peritoneum. 2025 Dec 15;11(1):11–8. doi: 10.1515/pp-2025-0034 (PMC13001815; doi:10.1515/pp-2025-0034)
Supplement: Supplementary file 1 — Supplementary Material [file j_pp-2025-0034_suppl_001.docx]

High prevalence of *KRAS* and *GNAS* mutations in pseudomyxoma peritonei underscores opportunities for targeted therapeutic strategies

Annette Torgunrud^1 *^, Christin Lund-Andersen^1 *^, Ben Davidson^2,3^ , Ina Katrine Nitschke Marcussen^1^, Vegar Dagenborg^4^ , Kjersti Flatmark ^1,2,4^

^1^Department of Tumor Biology, Norwegian Radium Hospital, Oslo University Hospital, Oslo, Norway

^2^Department of Pathology, Oslo University Hospital, Oslo, Norway

^3^Institute of Clinical Medicine, Medical Faculty, University of Oslo, Norway

^4^Department of Surgical Oncology, Norwegian Radium Hospital, Oslo University Hospital, Oslo, Norway

* Equal contribution

Corresponding author:

Annette Torgunrud, Department of Tumor Biology, Oslo University Hospital, Oslo, Norway

https://orcid.org/0000-0001-5751-9549

**Supplementary materials sections:**

**Figure S1:** Patient surgery trends (2002–2022)

**Table S1:** Microscopy assessment of fresh-frozen samples

**Table S2:** Cases with mutations other than *KRAS* and *GNAS*

**Table S3:** Multivariable analysis of survival parameters

**Supplementary tables and figures**


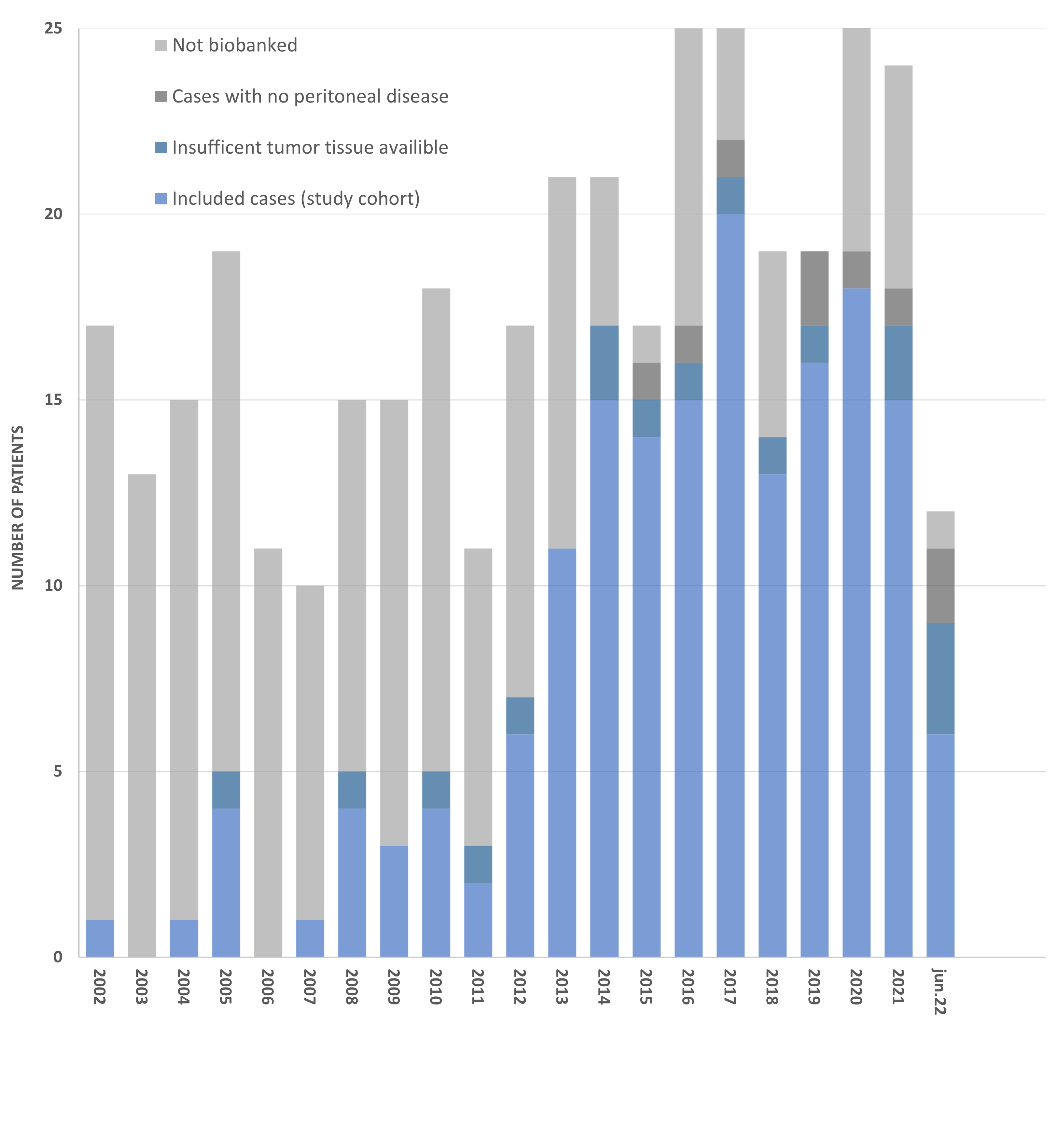


**FigureS1:** Number of patients undergoing surgery for PMP at the Norwegian Radium Hospital, Oslo University Hospital per year between 2002 and 2022 (n=296). Biobanking was limited up until 2013, and hampered in 2020 due to the Coronavirus pandemic. The light blue bars: cases analyzed and included in this study cohort (N=167). Grey bars: not biobanked (N=100). Dark grey: cases with no peritoneal disease (N=11). Dark insufficent tumor availible, and for one patient, insufficient DNA was retrieved from the sample (N=18).

**Table S1 Microscopy assessment of fresh-frozen samples**

|  | Final histopathological classification (PSOGI) of the surgical specimen from the CRS-HIPEC procedure | | | | |
| --- | --- | --- | --- | --- | --- |
| Tumor cell fraction determined by microscopy of frozen sections  % | *Acellular mucin* | *Low- grade* | *High- grade* | *High-grade with signetring cells* | Total # patients |
| 0 | 21 | 49 | 1 | 0 | 71 |
| <1 | 0 | 11 | 5 | 0 | 16 |
| 1-10 | 0 | 37 | 6 | 0 | 43 |
| 10-50 | 0 | 7 | 4 | 0 | 11 |
| >50 | 0 | 4 | 1 | 3 | 8 |
| Total # patients | 21 | 108 | 17 | 3 | 149 |

**Table S2** **The cases with detected mutations other than *KRAS* and *GNAS***

| *Patient* | *Appendix tumor* | *Peritoneal disease* | *KRAS* | *GNAS* | *Gene* | *Code* | *MAF %* |
| --- | --- | --- | --- | --- | --- | --- | --- |
| 1 | LAMN | LG | G12D | WT | *SMAD4* | E520X | 3 |
|  |  |  |  |  | *SMAD4* | P345L | 3 |
| 2 | LAMN | LG | G12V | R201H | *SMAD4* | R361H | 15 |
| 3 | Unknown | HG | G12C | R201C | *ATM* | R3008C | 6 |
| 4 | LAMN | LG | G12D | R201C | *AKT1* | E17L | 15 |
|  |  |  |  |  | *PIC3CA* | A1046T | 3 |
| 5 | LAMN | LG | wt | R201H | *ESR1** | R157Ter | 3.4 |
|  |  |  |  |  | *FANC1** | T937A | 4.6 |
| 6 | LAMN | acellular mucin | G12V | R201H | *Notch* | V175del | 1.3 |
| 7 | unknown | LG | G12V | R201C | *TP53* | Y220H | 8 |
| 8 | LAMN | LG | G12D | R201C | *SMAD4* | G365R | 4 |
|  |  |  |  |  | *SMAD4* | R361H | 4 |
| 9 | LAMN | LG | G13D | R201H | *IDH2** | R140Q | 2.7 |
|  |  |  |  |  | *PIC3CA* | A1046TW | 2.7 |
| 10 | Mucinous adenocarcinoma | HG | G12D | R201H | *NOTCH3* | D1086A | 3.2 |
| 11 | Unknown | LG | wt | wt | *PIC3CA* | A1046T | 4.6 |
| 12 | LAMN | LG | G12D | R201H | *CTNNB1* | S45F | 1.2 |
|  |  |  |  |  | *FBXW7* | A505C | 1.2 |
|  |  |  |  |  | *TP53* | H168Y | 1.3 |
| 13 | LAMN | LG | G12V | R201H | *ATM* | S2259T | 1 |
| 14 | LAMN | LG | wt | wt | *SF3B1** | L666N | 6.9 |
| 15 | Unknown | LG | wt | wt | *GNA11* | Q209L | 1.8 |
| 16 | HAMN | HG | wt | R201H | *BRAF* | V600E | 7.9 |
|  |  |  |  |  | *CDKN2A* | R58Q | 13.7 |
|  |  |  |  |  | *FBXW7* | R505C | 6.7 |
|  |  |  |  |  | *TP53* | R174H | 6 |
| 17 | HAMN | acellular mucin | G12D | R201H | *PIC3CA* | A1046T | 6.5 |
| 18 | LAMN | HG | G12V | R210H | *TP53* | R110H | 1.3 |
| 19 | Mucinous adenocarcinoma | HG | G12D | R201H | *FBXW7* | T446C | 6.6 |
| 20 | HAMN | HG with signet ring cells | wt | wt | *TP53* | R236Q | 14.6 |
| 21 | LAMN | LG | G12V | R201H | *FBXW7* | S582L | 6 |
|  |  |  |  |  | *SMAD4* | R445X | 6 |
| 22 | LAMN | LG | G13D | R201H | *BRAF* | S605N | 1.3 |
|  |  |  |  |  | *EGFR* | G735C | 1.4 |
|  |  |  |  |  | *PIC3CA* | G542A | 7.3 |
| 23 | HAMN | HG | G13D | R201S | *TP53* | D248Q | 15.4 |
| 24 | Mucinous adenocarcinoma | HG | G12V | wt | *SMAD4* | Y353S | 17 |
| 25 | HAMN | LG | G12V | R201H | *PIC3CA* | Q546R | 12.8 |
| 26 | LAMN | LG | G13D | R201H | *SMAD4* | L385Ter | 12.1 |
| 27 | Mucinous adenocarcinoma | HG | G12V | R201C | *AKT1* | E17L | 2.1 |
| 28 | Goblet cell carcinoid | HG with signet ring cells | wt | R201C | *SMAD4* | R497H | 2 |
| 29 | LAMN | LG | G12D | R201H | *SMAD4* | R361C | 6.7 |
| 30 | LAMN | acellular mucin | G12V | R201H | *Ret* | L923P | 3.6 |
| 31 | LAMN | LG | wt | wt | *EGFR* | G735V | 2 |
| 32 | LAMN | LG | wt | wt | *NRAS* | G13D | 8 |
| 33 | Mucinous adenocarcinoma | HG | G13D | wt | *PIC3CA* | H1047R | 22 |
|  |  |  |  |  | *SMAD4* | K392X | 43 |
| 34 | Unknown | HG | wt | R201C | *BRAF* | V600E | 25 |
|  |  |  |  |  | *CTNNB1* | D32G | 21 |
| 35 | LAMN | LG | wt | wt | *NRAS* | Q61H | 8 |

LAMN= low-grade appendiceal mucinous neoplasm group, HAMN= High-grade appendiceal mucinous neoplasms, HG= high-grade, LG= low-grade, MAF= mutated allele frequency, *mutations only detected with Oncomine Comprehensive Assay v3.

**TableS3: Multivariable analysis of significant parameters associated with overall survival**

|  | HR (95%CI) | p-value |
| --- | --- | --- |
| Peritoneal cancer index |  |  |
| Increasing | 1.04 (0.94-1.15 | 0.455 |
| Mutations |  |  |
| *SMAD4* | 12.66(0.08-2076) | 0.329 |
| Tumor markers |  |  |
| CA19-9 | 1.00 (1.00-1.01) | 0.03 |
| CA-125 | 1.01 (1.00-1.04) | 0.07 |

CA19-9 cancer antigen 19-9, Ca-125 cancer antigen 125
